# Supplementary material for: Increased Mild Vaccine-Related Side Effects and Higher Specific Antibody Titers in Health Care Workers with Previous SARS-CoV-2 Infection after the mRNA BNT162b2 Vaccine
Source: Vaccines (Basel). 2022 Aug 2;10(8):1238. doi: 10.3390/vaccines10081238 (PMC9414957; doi:10.3390/vaccines10081238)
Supplement: Supplementary file 1 [file vaccines-10-01238-s001.zip › vaccines-1768578-supplementary.pdf]

Supplemental Table S1. Number of side effects in the study population after the first vaccine dose, overall and stratified according to prior SARS-CoV-2 infection.

Number of side effects	First vaccine dose			
	Overall (n=1106)	Previous SARS-CoV-2 infection Yes (n=83)	No (n=1023)	*p*
0	354 (32.0%)	18 (21.7%)	336 (32.8%)	0.021
1	501 (45.3%)	36 (43.4%)	465 (45.5%)	
2	154 (13.9%)	16 (19.3%)	138 (13.5%)	
≥ 3	97 (8.8%)	13 (15.7%)	84 (8.2%)	

Supplemental Table S2. Number of side effects after the first and second vaccine doses in the study population after case-control matching, overall and stratified according to prior SARS-CoV-2 infection.

N° of side effects	First vaccine dose				Second vaccine dose			
		Previous SARS-CoV-2 infection				Previous SARS-CoV-2 infection		
	Overall (n=249)	Yes (n=83)	No (n=166)	*p*	Overall (n=239)	Yes (n=82)	No (n=157)	*p*
0	64 (25.7%)	18 (21.7%)	46 (27.7%)	0.028	50 (20.9%)	20 (24.4%)	30 (19.1%)	0.133
1	123 (49.4%)	36 (43.4%)	87 (52.4%)		68 (28.4%)	29 (35.4%)	39 (23.8%)	
2	40 (16.1%)	16 (19.3%)	24 (14.5%)		48 (20.1%)	14 (17.1%)	34 (21.6%)	
≥ 3	22 (8.8%)	13 (15.7%)	9 (5.4%)		73 (30.5%)	19 (23.2%)	54 (34.4%)	

Information on side effects after second vaccine dose is missing for 10 subjects (1 with prior infection and 9 without).
